# Development of Styrene-Grafted Polyurethane by Radiation-Based Techniques

**DOI:** 10.3390/ma9060441

**Published:** 2016-06-02

**Authors:** Jin-Oh Jeong, Jong-Seok Park, Youn-Mook Lim

**Affiliations:** Radiation Research Division for Industry and Environment, Korea Atomic Energy Research Institute, 1266 Sinjeong-dong, Jeongeup-si, Jeollabuk-do 580-185, Korea; jinoh0209@kaeri.re.kr (J.-O.J.); ymlim71@kaeri.re.kr (Y.-M.L.)

**Keywords:** polyurethane, styrene, grafting polymerization, gamma-irradiation

## Abstract

Polyurethane (PU) is the fifth most common polymer in the general consumer market, following Polypropylene (PP), Polyethylene (PE), Polyvinyl chloride (PVC), and Polystyrene (PS), and the most common polymer for thermosetting resins. In particular, polyurethane has excellent hardness and heat resistance, is a widely used material for electronic products and automotive parts, and can be used to create products of various physical properties, including rigid and flexible foams, films, and fibers. However, the use of polar polymer polyurethane as an impact modifier of non-polar polymers is limited due to poor combustion resistance and impact resistance. In this study, we used gamma irradiation at 25 and 50 kGy to introduce the styrene of hydrophobic monomer on the polyurethane as an impact modifier of the non-polar polymer. To verify grafted styrene, we confirmed the phenyl group of styrene at 690 cm^−1^ by Attenuated Total Reflection Fourier Transform Infrared Spectroscopy (ATR-FTIR) and at 6.4–6.8 ppm by ^1^H-Nuclear Magnetic Resonance (^1^H-NMR). Scanning Electron Microscope (SEM), X-ray Photoelectron Spectroscopy (XPS), Thermogravimetric Analysis (TGA) and contact angle analysis were also used to confirm styrene introduction. This study has confirmed the possibility of applying high-functional composite through radiation-based techniques.

## 1. Introduction

Due to their excellent physical properties and low price, polyolefin-based polymers have recently gained wide use in daily life. This class of general-purpose polymers forms the largest section of the consumer market, partly because they are commonly used in household goods and industrial structural materials [1,2,3]. Polyolefin-based polymer resins, such as Polypropylene (PP), Polyethylene (PE), Polyvinyl chloride (PVC), and Polystyrene (PS), have been most frequently used. Their excellent performance mechanical strength and workability make them quite versatile, but their low impact resistance is a disadvantage. Accordingly, studies are actively underway to improve this property [4].

In contrast, polyurethane (PU) has excellent impact resistance, mechanical strength, thermal stability, and chemical resistance. PU has been used in the production of flexible foams for beds, automobiles and sofas, rigid foams used as an insulating material of refrigerators and buildings, paints, adhesives, spandex fibers, and thermoplastic polyurethane [5,6,7]. PU is the fifth most commonly used polymer in consumer markets, following PP, PE, PVC, and PS, and is the most commonly used polymer in thermosetting resins. PU has excellent physical properties, such as elasticity and flexibility similar to rubber, and is relatively stable in the body. To improve heat resistance, blending of PU and polyolefin-based polymers was required [8]. These blended composites were developed for use in branching and to improve upon disadvantages of the individual polymers [9]. 

However, difficulties arise in the blending of hydrophobic polyolefin-based polymers and the hydrophilic polyurethane polymer. This blending reduces problems with mechanical strength and can be achieved using chemical additives or surfactants or polymer modification techniques [10].

The grafting polymerization reaction is a method of polymerizing a monomer onto a polymer used for branching a polymer chain onto a substrate polymer. The polymers made through grafting polymerization are durable and maintain their physical properties [11,12].

The typical polymer modification techniques use chemical modification, plasma, and radiation [13]. Disadvantages of chemical modification include long process time, complicated process, and required chemical additives, such as initiators and catalysts. Conversely, radiation technology, which works through formation of free radicals due to ionization of the materials and the monomers with gamma-irradiation and electron beam energy has the advantage that the monomers can be grafted without chemical additives [14,15]. In addition, radiation can be used in solid state or at low temperatures. Radiation can also be employed in a short period of time, which has the advantage of minimized energy consumption [16,17]. 

In this study, the blending of polyolefin-based polymers and PU was completed by grafting hydrophobic monomers of styrene onto PU using gamma irradiation. Polymer grafting was verified through characterization with ATR-FTIR, SEM, NMR, XPS, and TGA analysis. The possibility of high-performance composite materials using radiation techniques has been confirmed.

## 2. Experimental 

### 2.1. Materials

Methylene diphenyl diisocyanate (MDI)-based polyurethane (PU) was purchased from Songwon Industrial Co., Ltd. (Ulsan, Korea). The average molecular weight was approximately 80,000. Styrene monomer was purchased from Showa Chemical Co. (Tokyo, Japan). Methanol and tetrahydrofuran (THF) were purchased from Duksan reagent (Ansan, Korea). All other reagents and solvents were of analytical grade and were used as received.

### 2.2. Preparation of Styrene Grafted Polyurethane

**Figure 1:**
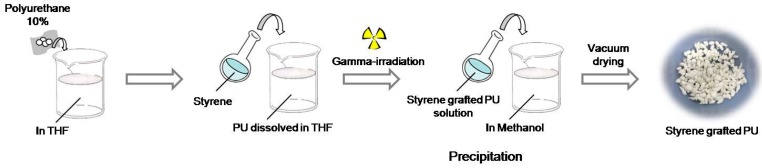
Schematic diagrams of styrene grafted polyurethane.

**Scheme 1:**
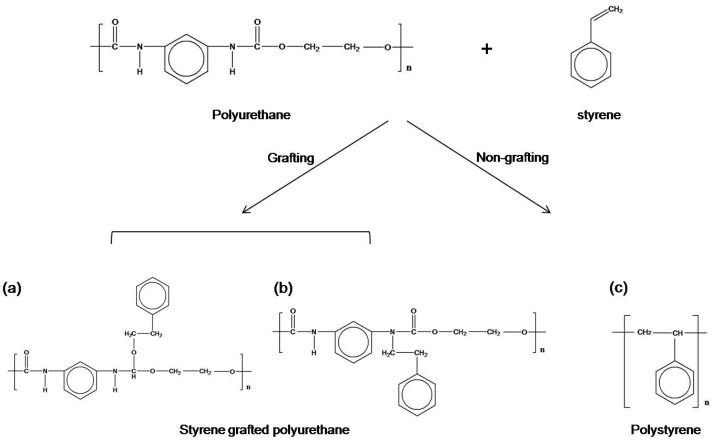
Radiation-induced grafting polymerization.

The 10 wt % PU was dissolved in THF solution at room temperature. Styrene monomer was later immersed in PU/THF solution at different concentrations (5 wt %, 10 wt %, and 20 wt %). The prepared mixtures were exposed to a gamma ^60^Co sources (ACEL type C-1882, Korea Atomic Energy Research Institute) with a total absorbed dose ranging from 25 to 50 kGy (10 kGy/h). To remove the remaining solvent, the PU was precipitated three times in methanol and subsequently vacuum-dried for 48 h. The overall pattern diagrams are shown on Figure 1 and Scheme 1. The characteristics of styrene-grafted polyurethane are shown in Table 1.

### 2.3. Attenuated Total Reflection Fourier Transform Infrared Spectroscopy (ATR-FTIR)

Infrared spectra of the PU and styrene grafted PU (SPU) were recorded using Bruker TEMSOR 37 (Bruker AXS. Inc., Karlsruhe, Germany) equipped with ATR mode over the range of 650 to 4000 cm^−1^ at a resolution of 6 cm^−1^ averaged over 64 scans. The conversion rate of phenyl was calculated using the following equation:

(1) $$\mathrm{Conversion}\mathrm{rate}\left(\%\right)=\left[1-\left(P_{u}-P_{m}\right)\right]\times 100\%$$

where *P*_u_ and *P*_m_ represent the peak intensity (670–725 cm^−1^) of unmodified PU and modified PU, respectively.

### 2.4. Scanning Electron Microscope (SEM)

To observe the high-resolution images of the PU and SPU with increasing the concentration of styrene and irradiation dose, the polymers were coated with a layer of gold for 60 seconds by sputter coating. The morphology of the polymers was investigated using SEM (JSM-6390, JEOL, Tokyo, Japan) with an electron beam of 10 kV and a working distance of 10 to 12 mm. 

### 2.5. Blue Ink Staining

The samples stained with slow shaking in a 24 well plate containing 1 mL of water-based blue ink (PILOT, 30cc, Seongnam, Korea) for 4 h at room temperature. Each sample was washed three times for 12 h in deionized water (DIW).

### 2.6. ^1^H-Nuclear Magnetic Resonance (^1^H-NMR)

The structure of PU and SPU were determined by 500 MHz ^1^H-NMR (ECA 500 MHz spectrometer, JEOL, Tokyo, Japan) with averaged over 32 scans. Next, 3.5 mg PU and SPU were dissolved in 0.5 mL THF-d_8_ solution, and product spectra were obtained.

### 2.7. X-ray Photoelectron Spectroscopy (XPS)

Analysis of PU and SPU was performed on X-ray photoelectron spectra (XPS, Theta Probe AR-XPS System, Thermo Fisher Scientific, Waltham, MA, USA) fitted with an Al Kα source (soft X-ray source at 1486.6 eV, which is monochromated). The anode was operated at 150 W (15 kV).

### 2.8. Thermogravimetric Analysis (TGA)

Thermogravimetric analysis was performed using TA Q600 (TA Instrument, New Castle, PA, USA). Next, 15.0 mg PU and SPU were prepared and placed in a platinum pan. The analysis was performed at a heating rate 10 °C/min from 40 to 700 °C under nitrogen flow.

### 2.9. Contact Angle

The static water contact angles were measured according to the sessile drop method using a Phoenix 300 contact angle analyzer (Surface Electro Optics Co., Suwon, Korea). Regarding the water contact angle, at least five specimens were tested, and the average value was taken. 

## 3. Results and Discussion

As shown in Scheme 1, it is possible to infer two possible routes of the styrene grafting onto PU via radiation. Initially, the double bond of the styrene was broken by radiation, causing radical formation in the branch and styrene grafting to the carbonyl group of PU. The second reaction demonstrates how styrene could be grafted onto the amine group of PU, whereas polystyrene could be produced by free radical polymerization, from non-grafted styrene.

**Figure 2:**
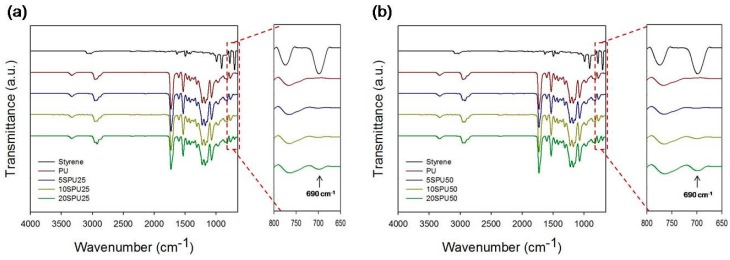
Surface chemical properties of styrene grafted polyurethane by Attenuated total reflectance-Fourier transform infrared spectroscopy (ATR-FTIR) at dose rate: (**a**) 25 kGy; and (**b**) 50 kGy.

Figure 2 illustrates the chemical properties of PU and SPU composites investigated by ATR-FTIR analysis. As shown in the SPU spectra, a new signal from a phenyl group was observed at 690 cm^−1^ due to grafting polymerization of styrene on the PU. In addition, the intensity of peak was significantly increased with increases in the concentration of styrene and radiation dose. Table 2 shows that the conversion rate of phenyl group was 56.6% in the 5 wt % styrene-grafted PU, whereas with 20 wt % styrene-grafted PU, it increased to 82.8% under a radiation dose of 25 kGy. The conversion rate of styrene-grafted PU at a radiation dose of 50 kGy also increased. The styrene content increases with the conversion rate of the phenyl group.

**Figure 3:**
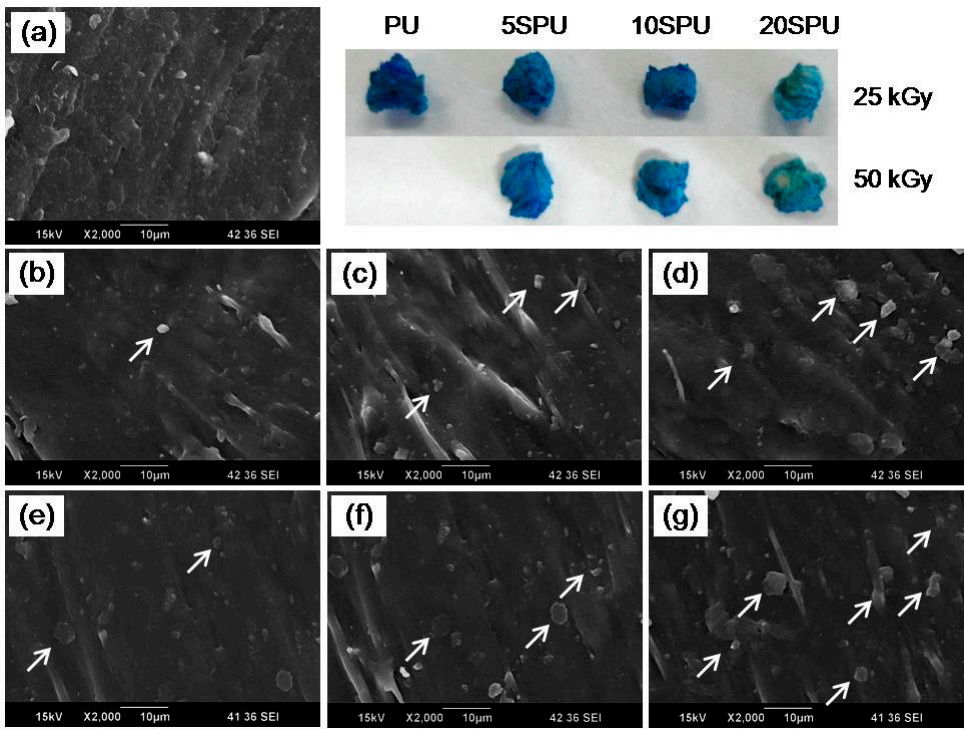
Scanning electron microscopy (SEM) images of (**a**) polyurethane, styrene grafted polyurethane dose rate at 25 kGy; (**b**) 5 wt %; (**c**) 10 wt %; (**d**) 20 wt %; dose rate at 50 kGy; (**e**) 5 wt %; (**f**) 10 wt %; and (**g**) 20 wt %.

Figure 3 shows the SEM micrographs of surface morphologies of PU and SPU composites at different styrene contents and radiation doses. The surface morphologies of unmodified PU were almost smooth but became rougher with increasing styrene content and radiation dose [5,6]. The surface morphology of the SPU composites were dependent on the styrene content and radiation dose [18,19]. Unmodified PU was stained blue with increasing styrene content, while the SPU was only stained light blue due to presence of hydrophobic styrene groups.

**Figure 4:**
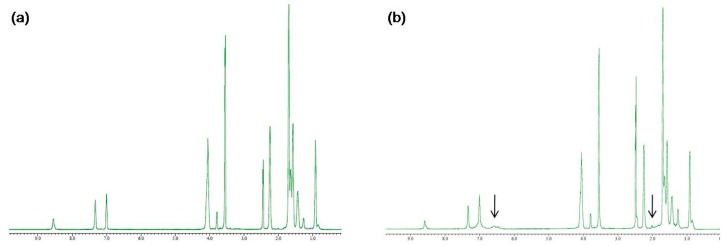
^1^H-Nuclear magnetic resonance (^1^H-NMR) spectra of (**a**) polyurethane; and (**b**) 20% styrene grafted polyurethane at 50 kGy.

Figure 4 shows the ^1^H-NMR spectrum of PU and 20SPU50. The peaks at 4.1 and 2.6 ppm corresponded to the characteristic methylene protons of COOCH_2_CH_2_, respectively. Additionally, the peak at 3.6 ppm assigned to the methyl proton of OCH_3_ was observed [20]. After grafting, the peak of aromatic ring of styrene at 6.4–6.8 ppm was observed [21,22]. Moreover, the peak of methylene protons of styrene appeared at 2.1 ppm. This result suggest that the SPU has been successfully grafted. 

**Figure 5:**
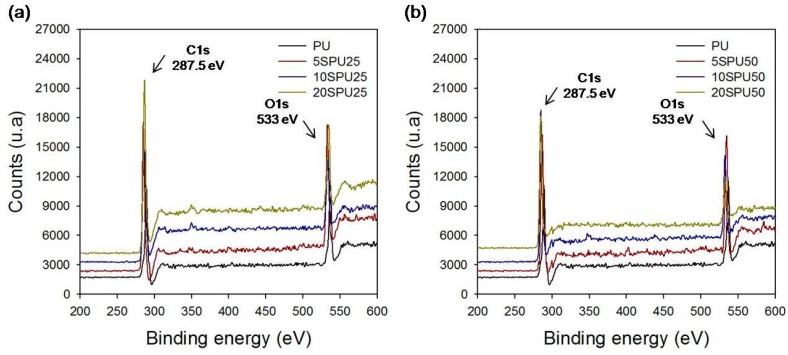
X-ray photoelectron spectroscopy (XPS) spectra of styrene grafted polyurethane at dose rate: (**a**) 25 kGy; and (**b**) 50 kGy.

The XPS spectra of the PU and SPU are shown in Figure 5 according to styrene content. The strong carbon peak together with oxygen peak of PU and SPU indicated binding energies of 287.5 and 533 eV, respectively [6]. The atomic concentrations with the styrene content and radiation dose are presented in Table 3. The carbon and oxygen content of PU were found to be 76.4% and 21.2%, respectively. Carbon content was significantly increased after styrene grafting; it was found to be 78.1%, 80.1% and 81.1% for the 5 wt %, 10 wt %, and 20 wt % styrene reacted under a radiation dose of 25 kGy. Conversely, the oxygen content was found to be 19.6%, 17.0%, and 15.4%, respectively, indicating that the hydrophobicity of SPU was significantly increased due to the radiation-induced styrene grafting. It was possible to verify that the carbon content is higher and composed of a hydrophobic structure [14,23]. The carbon content of styrene grafted PU at 50 kGy also increased, but oxygen content decreased at 25 kGy. The amount of carbon in 20SPU50 increased by 6.2% more than for 20SPU25, but the amount of oxygen decreased by 4.9%. This finding is most likely observed due to grafting of styrene into the carbonyl group of polyurethane through radiation.

**Figure 6:**
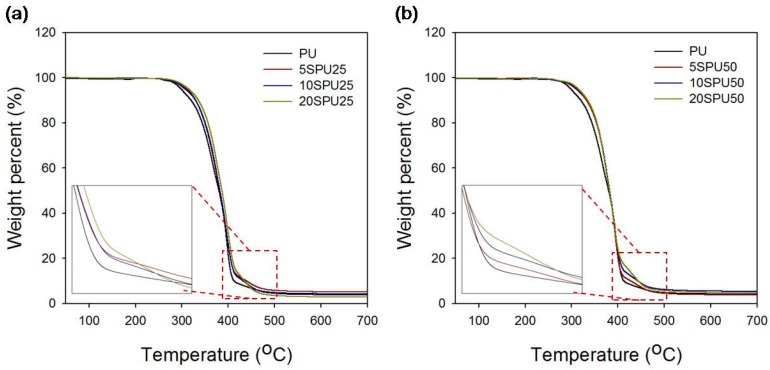
Thermogravimetric analysis (TGA) curves of styrene grafted polyurethane at dose rate: (**a**) 25 kGy; and (**b**) 50 kGy.

As shown in Figure 6 and Table 4, the thermal stability of the PU and SPU was determined by TGA. At the initial degradation temperature of approximately 300 °C, when the styrene content was increased, thermal stability was also increased [24]. In addition, the new degradation peaks in SPU composites were obviously observed at 400–460 °C due to the decomposition of styrene [21]. Remaining PU content was 11.0% at 410 °C, and the remaining amount of SPU confirmed for 5 wt %, 10 wt %, and 20 wt % styrene content was 14.9%, 15.1%, and 17.8% at 25 kGy, respectively. In addition, when the radiation dose was 50 kGy, the remaining SPU content was 12.4%, 15.2%, and 17.8%, respectively. Thermal stability increased as styrene content increased, most likely due to the excellent heat resistance and machinability of styrene.

**Figure 7:**
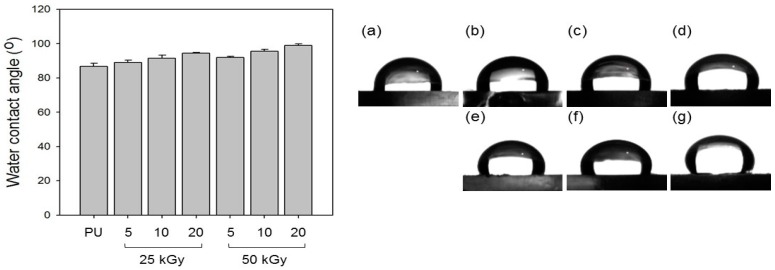
Contact angles of (**a**) polyurethane, styrene grafted polyurethane dose rate at 25 kGy; (**b**) 5 wt %; (**c**) 10 wt %; (**d**) 20 wt %; dose rate at 50 kGy; (**e**) 5 wt %; (**f**) 10 wt %; and (**g**) 20 wt %.

Comparison of the water contact angles of PU and SPU can reveal changes in the hydrophobic characterization on the PU that was grafted to the PS after the radiation-induced grafting. Figure 7 shows the water contact angles of PU and SPU composites at different styrene contents and radiation doses. The water contact angle increased for increasing the radiation doses and the styrene content. As shown in Figure 7, the water contact angle of unmodified PU was 86.6°, whereas at 25 kGy, the water contact angle of SPU for 5 wt %, 10 wt %, and 20 wt % was 88.8°, 91.4°, and 94.3°, respectively. In addition, when the radiation dose was 50 kGy, the water contact angle of SPU for 5 wt %, 10 wt %, and 20 wt % was 91.9°, 95.5°, and 98.8°, respectively. It can be attributed to the higher hydrophobicity of the PS due to the radiation-induced grafting. 

## 4. Conclusions

In this study, to modify a hydrophilic structure into hydrophobic structure, styrene-grafted PU was generated by radiation-based techniques. When the absorbed styrene content was 20 wt % and radiation dose was 50 kGy, the surface morphology of the SPU was rough. According to NMR, the carbonyl group of SPU peak intensity is decreased compared to PU, whereas the benzylic group peak intensity is increased. It was established that styrene was grafted to carbonyl group of PU. SPU has a hydrophobic structure due to increased presence of benzylic groups. The possibility of applying high-functional composites by radiation-based techniques has been confirmed.

## Figures and Tables

**Table 1:** Characteristics of styrene-grafted polyurethane.

Samples	Concentration of Styrene (%)	Irradiation Dose (kGy)
5SPU25	5	25
10SPU25	10	25
20SPU25	20	25
5SPU50	5	50
10SPU50	10	50
20SPU50	20	50

**Table 2:** Conversion rate of aromatic (phenyl ring) of SPU from ATR-FTIR.

Samples	Conversion Rate (%)
5SPU25	56.6
10SPU25	60.6
20SPU25	82.8
5SPU50	59.8
10SPU50	74.4
20SPU50	87.1

**Table 3:** Atomic concentration with the styrene content and radiation dose.

Samples	Carbon (%)	Oxygen (%)
PU	76.4	21.2
5SPU25	78.1	19.6
10SPU25	80.1	17.0
20SPU25	81.1	15.4
5SPU50	77.6	20.3
10SPU50	80.5	16.5
20SPU50	87.4	10.6

**Table 4:** Remaining amounts (410 °C) of SPU from TGA.

Samples	Weight Percent (%)
PU	11.0
5SPU25	14.9
10SPU25	15.1
20SPU25	17.8
5SPU50	12.4
10SPU50	15.2
20SPU50	17.8
